# Current research status and hotspots of precancerous lesions of gastric cancer: a bibliometric analysis

**DOI:** 10.3389/fonc.2025.1571617

**Published:** 2025-04-29

**Authors:** Yan Huang, Dexin Wang, Yuzhuo Liu, Xiaonan Xu, Li Chen, Zhuolin Ma, Zhaoxia Liu

**Affiliations:** ^1^ First Affiliated Hospital of Heilongjiang University of Chinese Medicine, Harbin, China; ^2^ Shuguang Hospital Affiliated to Shanghai University of Traditional Chinese Medicine (TCM), Institute of Liver Diseases, Cell Biology Laboratory, Shanghai University of TCM, Key Laboratory of Liver and Kidney Disease of the Ministry of Education, Clinical Key Laboratory of TCM of Shanghai, Shanghai, China; ^3^ Second Affiliated Hospital of Liaoning University of Traditional Chinese Medicine, Shenyang, China; ^4^ Neonatology Department, Qiqihar Traditional Chinese Medicine Hospital, Qiqihar, China; ^5^ Heilongjiang Eye Hospital, Harbin, China

**Keywords:** precancerous lesions of gastric cancer, bibliometric, VOSviewer, Citespace, *Helicobacter pylori*

## Abstract

**Background:**

Gastric cancer is the fifth most common cancer worldwide, and its lack of specific symptoms presents a significant challenge for early diagnosis. Therefore, the identification and detection of precancerous lesions of gastric cancer (PLGC) are essential for its prevention. We performed a comprehensive bibliometric analysis to explore the research trends and emerging topics in this field, aiming to deepen our understanding of PLGC.

**Objective:**

This study utilizes a bibliometric approach with network analysis to explore the progress and trends in PLGC research. The findings aim to provide a foundation and guidance for further in-depth investigations into PLGC.

**Methods:**

This study used VOSviewer and CiteSpace software to collect relevant literature on PLGC from the Web of Science Core Collection, covering the period from 2005 to 2024. Data visualization analysis was performed on the number of publications, countries, institutions, journals, authors, keywords, and citation counts of these articles.

**Results:**

A total of 1,141 relevant articles were included in the analysis. The results showed a year-on-year increase in the number of publications from 2005 to 2024. The country, institution, author, and journal with the highest publication output in this field were China, Peking University, Wei-Cheng You, and *World Journal of Gastroenterology*, respectively. The most frequently occurring keywords in the PLGC field were “Helicobacter pylori,” “intestinal metaplasia,” “risk,” “infection,” and “atrophic gastritis.” Additionally, “chronic atrophic gastritis” and “inflammation” have emerged as hot topics for future research.

**Conclusion:**

This bibliometric analysis highlights the hot topics and emerging trends in PLGC research, aiming to provide valuable guidance for future studies. Our findings indicate that mechanistic studies and clinical diagnosis will be key areas of focus in upcoming research.

## 1 Introduction

Gastric cancer (GC) continues to be one of the leading causes of cancer-related deaths globally, with notably high incidence rates observed in East Asia, Eastern Europe, and certain regions of South America (1). Despite advances in treatment and early detection techniques, the prognosis for gastric cancer remains poor, largely due to its diagnosis at advanced stages. Consequently, the identification and monitoring of precancerous lesions of gastric cancer (PLGC) are vital for reducing mortality and enhancing survival rates through timely intervention (2). Precancerous lesions of gastric cancer represent a spectrum of histological changes that precede malignancy, including chronic atrophic gastritis, intestinal metaplasia, and dysplasia. These lesions offer critical opportunities for early intervention, particularly in high-risk populations, to prevent the progression to invasive cancer (3). The progression from precancerous lesions to gastric cancer is a complex process influenced by genetic, epigenetic, and environmental factors. Consequently, research on PLGC has gained significant momentum in recent years, with a focus on uncovering molecular pathways, identifying risk factors, and developing potential biomarkers for early diagnosis and prevention (4).

Bibliometric analysis has emerged as a powerful tool for assessing the current state of research and identifying emerging trends across various disciplines, including oncology and gastroenterology (5). Bibliometric analysis leverages the evaluation of scientific publications to quantify research productivity, identify influential studies, and map the knowledge landscape of specific topics. In the context of PLGC, bibliometric analysis provides valuable insights into the evolution and focal areas of this field, shedding light on research hotspots and uncovering potential knowledge gaps. Understanding these trends is crucial for guiding future research directions, optimizing resource allocation, and establishing clinical priorities in the prevention and treatment of gastric cancer (6).

This study performs a bibliometric analysis of research on PLGC, aiming to provide a comprehensive overview of the current research landscape and highlight emerging hotspots. By analyzing publication trends, leading authors, and highly cited works in the field, this study seeks to map the academic landscape of PLGC research and offer guidance for future studies focused on gastric cancer prevention and early detection.

## 2 Data and methods

### 2.1 Data source

**Figure 1:**
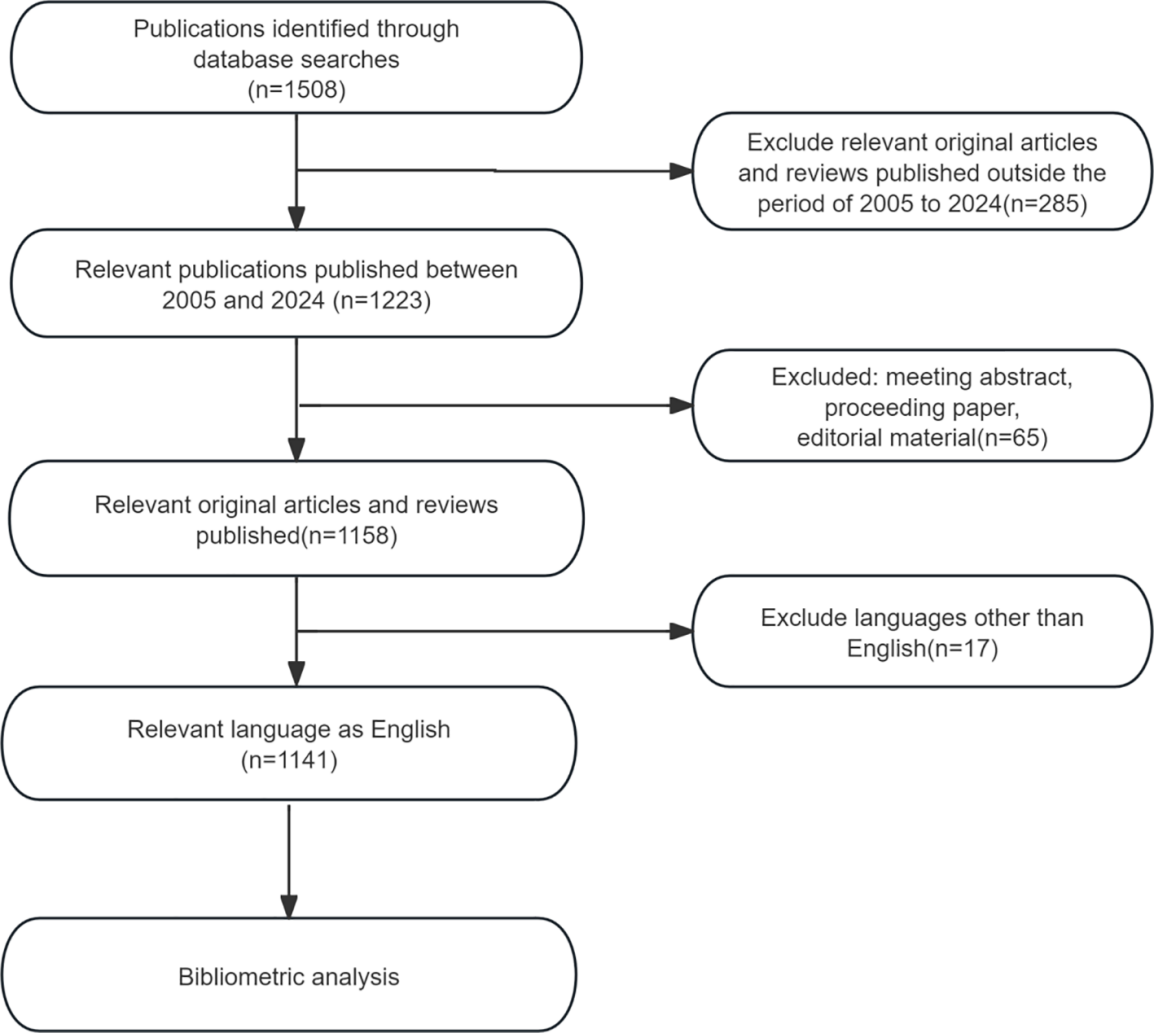
The inclusion and exclusion of publications on PLGC.

Considering factors such as coverage, citation frequency, and reliability, the Web of Science Core Collection (WoSCC) was selected as the data source for this study. WoSCC is renowned for its high-quality journals, standardized data, and support for interdisciplinary research, making it one of the most comprehensive and authoritative databases for bibliometric analysis, especially in the fields of natural sciences and medicine. The search formula used was Topic = “Precancerous Lesions of Gastric Cancer”. To explore the latest developments in this field, the search was conducted with a creation date up to March 24, 2025, covering documents from January 2005 to December 2024. The search was restricted to English-language publications, and the document types were limited to articles and review articles. The flowchart for the document selection process is presented in **Figure 1**.

### 2.2 Data processing

All selected literature was exported in plain text format and imported into Microsoft Excel 2019 to construct the bibliometric database. The analysis was performed using VOSviewer and CiteSpace, focusing on various aspects such as countries, institutions, authors, journals, references, and keywords from the selected literature. Several bibliometric indicators were employed for data analysis, including Average Citation Impact (ACI), H-index, centrality, and Sum of Times Cited (SOTC). These indicators are widely used to evaluate the impact of academic contributions. ACI represents the average number of citations per publication by a researcher, journal, or institution.

The H-index, which combines publication quantity and citation count, serves as a measure of academic influence for researchers, institutions, or journals. Centrality reflects the prominence of a paper or researcher within the citation network, indicating their importance in connecting various research outputs. SOTC refers to the total number of citations received by the entire body of work of a research entity over a specified period. These metrics collectively provide a comprehensive understanding of the academic impact and influence of the selected literature.

In the methods section, we have provided a more detailed explanation of the criteria and process for keyword selection and literature screening to ensure the accuracy and reliability of the data. Specifically, a keyword list was developed based on core terms defined in authoritative publications within the field, and the screening strategy was optimized using thematic search and Boolean logic operations to maximize the inclusion of relevant studies. While standardizing the screening steps, strict adherence to criteria such as the publication time range, language restrictions, and database sources was maintained to ensure scientific rigor. Additionally, when employing software tools such as VOSviewer and CiteSpace for data analysis, we explicitly described the details of parameter settings, including the choice of clustering algorithms, the determination of node thresholds, and the adjustment of time windows. These settings are crucial for the stability and scientific validity of the analytic outcomes. By implementing these measures, the transparency of the research methods has been significantly enhanced, thereby further ensuring the reliability and reproducibility of the results.

## 3 Results

### 3.1 Overview of the research

**Figure 2:**
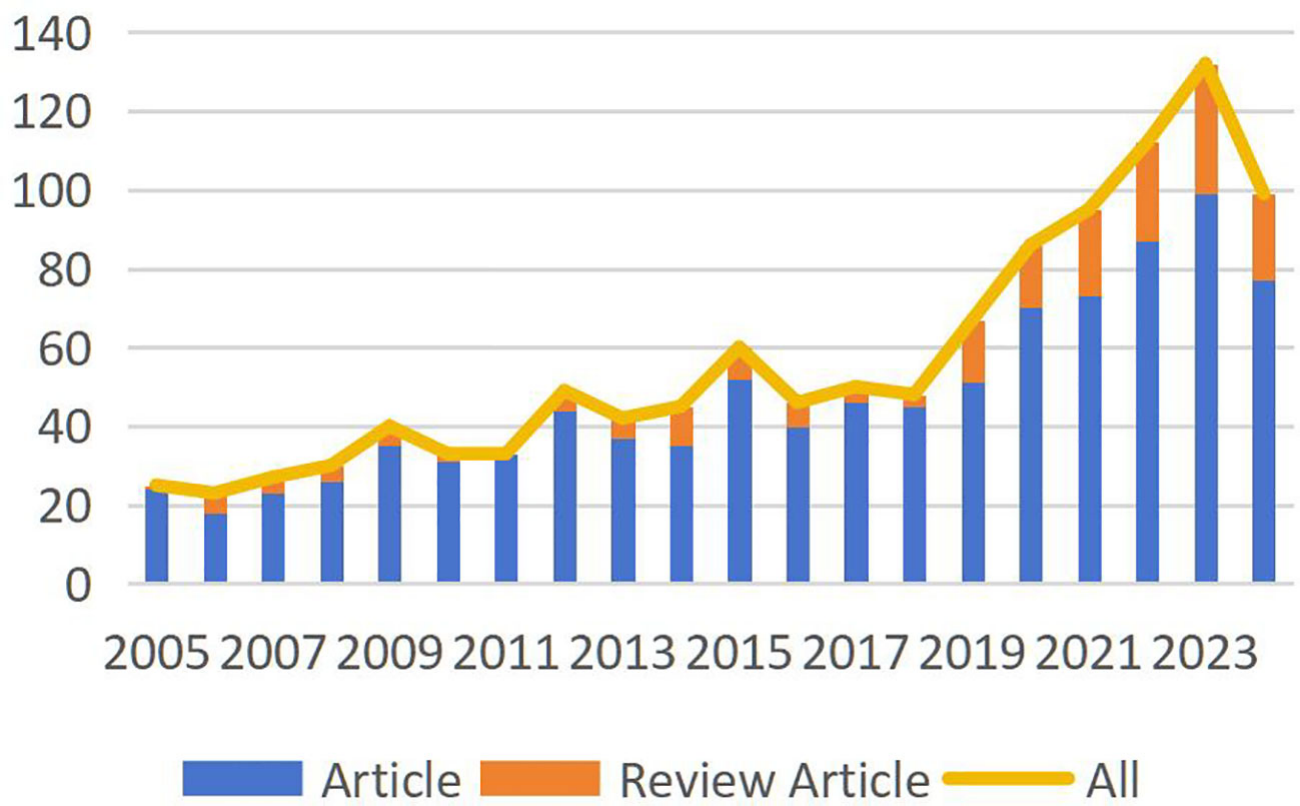
Trends of the related annual publications.

A total of 1,508 publications met the search criteria for this study, of which 1,141 articles were ultimately included after screening. These publications represent the collective contributions of 5,826 researchers from 74 countries or regions. **Figure 2** depicts the publication volume in this field over the past 20 years. Since 2018, there has been a consistent year-over-year increase in the number of published articles. Although data for 2024 only accounts for publications up to October, an overall upward trend in publication volume remains evident.

### 3.2 Country/region analysis

To evaluate the contributions of different countries and institutions in the field of PLGC, this study analyzed research publications from 1,058 institutions across 74 countries or regions. China emerged as the leading contributor, accounting for 49.87% of the total publications, nearly half of the global output. This was followed by the USA (15.34%), Japan (7.98%), and Italy (6.75%), while contributions from other countries each accounted for 4.73% or less (**Table 1**).

**Table 1:** Top 10 productive countries.

Rank	Country	Quantity	Proportion (%)	ACI	H-index	Centrality
1	China	569	49.87%	15.40	42	0.06
2	USA	175	15.34%	44.18	44	0.51
3	Japan	91	7.98%	43.31	32	0.08
4	Italy	77	6.75%	35.81	27	0.13
5	Germany	54	4.73%	40.70	24	0.05
6	South korea	54	4.73%	20.37	19	0.00
7	France	46	4.03%	47.63	18	0.01
8	Portugal	41	3.59%	60.10	23	0.00
9	Netherlands	36	3.16%	72.81	18	0.05
10	Iran	32	2.80%	16.09	14	0.10

**Figure 3:**
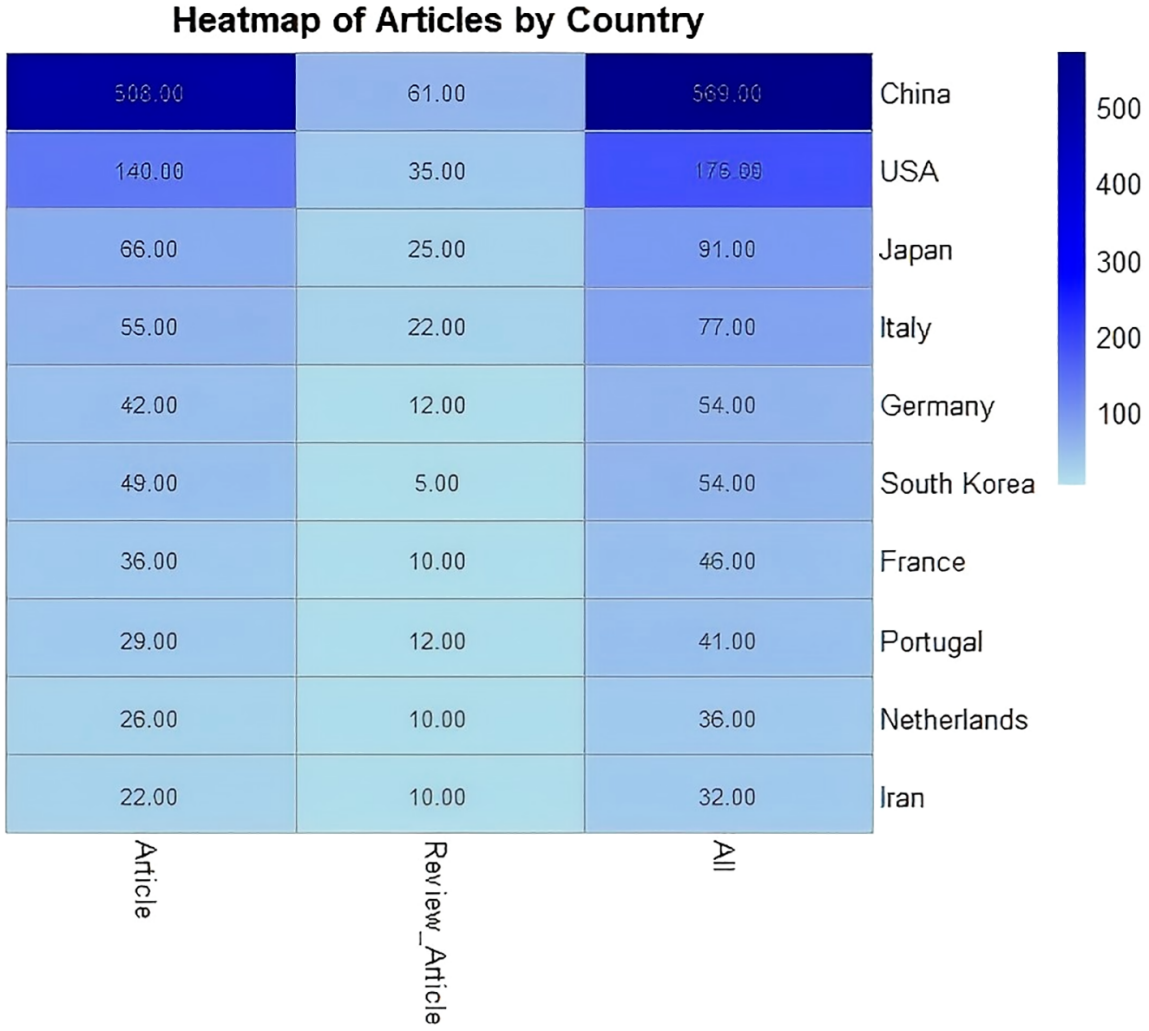
Heatmap of Articles by Country.

This heatmap shows the distribution of research and review articles across countries. China leads with 508 research articles and 569 total submissions, followed by the U.S. with 175 total articles, mainly research-focused. Japan and Italy contribute 91 and 77 articles respectively, with review articles making up a smaller portion. Germany and South Korea each have 54 submissions, while France, Portugal, the Netherlands, and Iran contribute fewer but are still research-focused. Overall, China and the U.S. dominate in both quantity and type, highlighting global disparities in scientific contributions (**Figure 3**).

**Figure 4:**
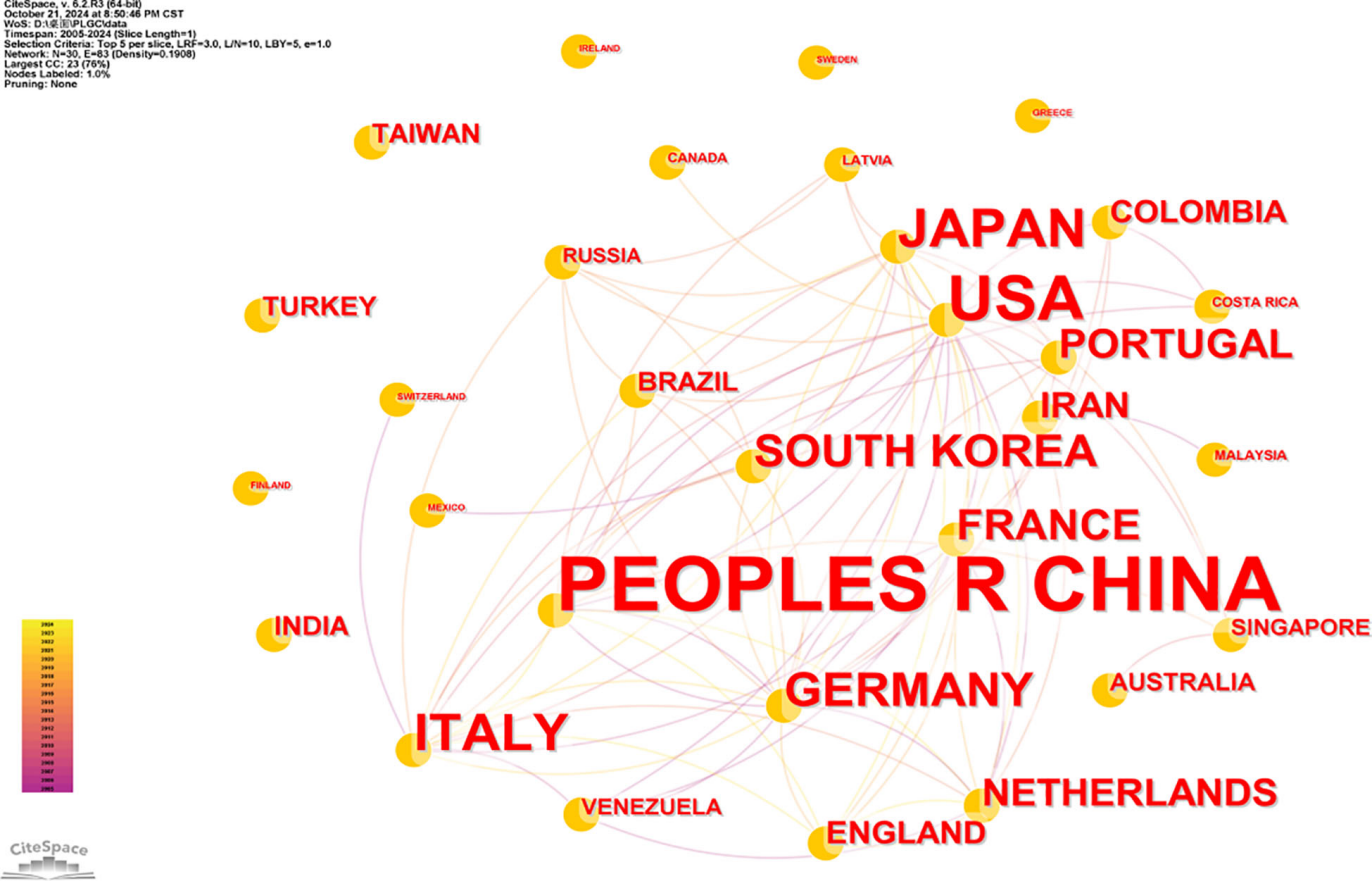
Collaboration visualization map of country/region.

To visualize collaboration between countries or regions, CiteSpace was used to map the top 30 contributing countries or regions. In **Figure 4**, the font size of each country’s name reflects its level of contribution, with larger font sizes indicating higher publication outputs. Notably, although China leads in PLGC publication volume, its ACI per paper is comparatively low compared to countries such as the Netherlands (72.81), Portugal (60.10), and France (47.63), whose publications have achieved higher academic recognition. Centrality analysis highlights the USA as a central hub within the PLGC research network, showing strong collaborative ties among various countries and regions.

### 3.3 Institutional distribution analysis

**Figure 5:**
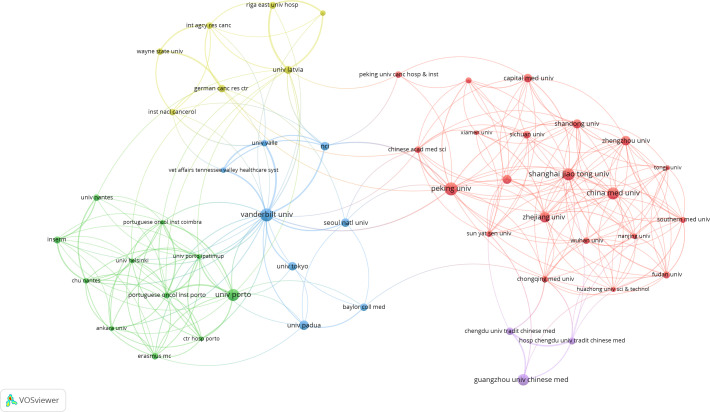
Collaboration visualization map of institutions.

A total of 1,058 institutions have conducted research on PLGC. Peking University in China has the highest publication output, with 50 papers, followed by Universidade do Porto in Portugal and Vanderbilt University in the USA. Shanghai Jiao Tong University and Guangzhou University of Chinese Medicine, both in China, rank fourth and fifth, with 33 and 31 publications, respectively. The top 10 institutions include several from China, as shown in **Table 2**. An institutional co-occurrence analysis illustrates the collaborative relationships among organizations. In **Figure 5**, node size represents the volume of publications, while node color intensity reflects the level of collaboration with other institutions. The top 50 institutions are listed, highlighting five major clusters represented by Peking University, Vanderbilt University, Universidade do Porto, Guangzhou University of Chinese Medicine, and University of Latvia. Strong collaborative ties are observed within each cluster.

**Table 2:** Top 10 productive institutions.

Rank	Institution	Country	Quantity	SOTC	ACI	H-index
1	Peking University	China	50	1832	36.64	21
2	Universidade do Porto	Portugal	36	2410	66.94	22
3	Vanderbilt University	USA	36	3104	86.22	20
4	Shanghai Jiao Tong University	China	33	653	19.79	13
5	Guangzhou University of Chinese Medicine	China	31	392	12.65	11
6	China Medical University	China	30	647	21.57	14
7	Zhejiang University	China	25	379	15.16	9
8	Chinese Academy of Medical Sciences Peking Union Medical College	China	24	314	13.08	10
9	US Department of Veterans Affairs	USA	24	818	34.08	14
10	Veterans Health Administration	USA	24	818	34.08	14

### 3.4 Author analysis

**Figure 6:**
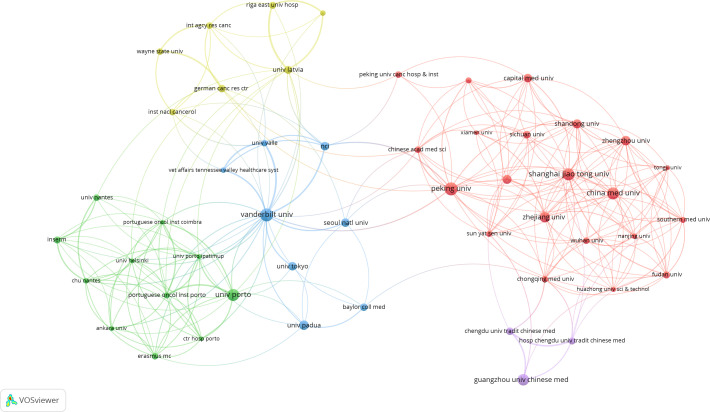
Analysis of author collaborations.

A total of 5,826 researchers have contributed to PLGC studies, with an average of 5.11 authors per paper. Notably, the two most prolific authors are from China: You Weicheng, with 35 publications, and Pan Kai-Feng, with 27 publications. Other leading authors include Dinis-Ribeiro Mario (n=24), Pan Huafeng (n=23), and Ma Junling (n=22). Among the top ten authors, four are from China, two from the USA, and one each from Portugal, Canada, Latvia, and Italy (**Table 3**). Authors with fewer publications, such as Correa Pelayo (79.94 ACI), Dinis-Ribeiro Mario (77.29), Zhou Tong (76.81), and Piazuelo M. Blanca (72.00), demonstrate relatively high ACI values, reflecting a greater overall impact of their work. **Figure 6** presents an author co-occurrence network, providing insights into collaborative relationships among researchers. Larger nodes indicate higher publication counts, while connecting lines signify collaborations between authors. The network reveals nine major working groups, each including four or more key authors, underscoring active collaboration within the field.

**Table 3:** Top 10 authors.

Rank	Author	Country	Institution	Quantity	ACI	H-index
1	You, Weicheng	China	Peking Univ Canc Hosp & Inst	35	41.91	19
2	Pan, Kai-Feng	China	Peking University	27	49.85	17
3	Dinis-Ribeiro, Mario	Portugal	Universidade do Porto	24	77.29	15
4	Pan, Huafeng	China	Guangzhou University of Chinese Medicine	23	12.13	10
5	Ma, Junling	Canada	University of Victoria	22	54.05	14
6	Piazuelo, M. Blanca	USA	Vanderbilt University	19	72.00	15
7	Correa, Pelayo	USA	Vanderbilt University	17	79.94	15
8	Zhou, Tong	Latvia	University of Latvia	17	14.59	9
9	Leja, Marcis	China	Peking Univ Canc Hosp & Inst	16	76.81	10
10	Annibale, Bruno	Italy	Sapienza University of Rome	13	63.31	9

### 3.5 Journal publication analysis

**Table 4** lists the top 20 journals with the highest number of publications on PLGC. The journal with the most articles is World Journal of Gastroenterology, with 44 publications, followed by Helicobacter (n=22), International Journal of Cancer (n=22), World Journal of Gastrointestinal Oncology (n=17), and Oncology Letters (n=16). The journal with the highest impact factor (IF) is Gastroenterology, boasting an IF of 25.70. In terms of journal rankings, the top three journals are all classified as Q1 journals. Among the top 20 journals, 14 fall into the Q1 and Q2 categories, while the remaining six belong to the Q3 category. Notably, Gastroenterology (average citations: 97.00), Journal of Digestive Diseases (51.83), and World Journal of Gastroenterology (41.25) exhibit the highest average citation counts, highlighting their influence and central role in advancing research within the PLGC field.

**Table 4:** Top 20 journals.

Rank	Journal	Quantity	ACI	IF (2023)	JCR
1	World Journal of Gastroenterology	44	41.25	4.30	Q1
2	Helicobacter	22	31.64	4.30	Q1
3	International Journal of Cancer	22	33.77	5.70	Q1
4	World Journal of Gastrointestinal Oncology	17	30.24	3.00	Q2
5	Oncology Letters	16	11.25	2.50	Q3
6	BMC Gastroenterology	14	20.14	2.50	Q2
7	Cancer Epidemiology Biomarkers & Prevention	14	37.00	3.70	Q1
8	European Journal of Gastroenterology & Hepatology	14	33.36	2.30	Q3
9	PLOS ONE	13	17.69	2.90	Q1
10	Digestive and liver disease	12	31.17	4.00	Q1
11	Digestive Diseases and Sciences	12	16.83	2.50	Q2
12	Frontiers in Pharmacology	12	7.33	4.40	Q1
13	Gastroenterology	12	97.00	25.70	Q1
14	Journal of Digestive Diseases	12	51.83	2.30	Q3
15	Medicine	12	3.50	1.30	Q2
16	Scandinavian Journal of Gastroenterology	12	23.58	1.60	Q3
17	Scientific Reports	12	24.92	3.80	Q1
18	Evidence-Based Complementary and Alternative Medicine	11	12.73	–	–
19	Journal of Ethnopharmacology	11	12.45	4.80	Q1
20	Asian Pacific Journal of Cancer Prevention	10	13.50	–	–

### 3.6 Analysis of hot topics and emerging trends

**Table 5** lists the top 20 most frequently occurring keywords, analyzed using CiteSpace. In addition to keywords directly associated with PLGC, terms such as “Helicobacter pylori,” “Intestinal metaplasia,” “Risk,” “Helicobacter pylori infection,” and “Atrophic gastritis” appeared with notable frequency. Keywords like “Helicobacter pylori,” “Intestinal metaplasia,” and “Atrophic gastritis” are closely linked to the pathogenesis of PLGC. Meanwhile, terms such as “Expression,” “Carcinogenesis,” and “Gene” are related to studies on mechanisms underlying PLGC development. Furthermore, keywords including “Risk,” “Diagnosis,” “Infection,” and “Classification” reflect aspects pertinent to the clinical diagnosis and management of PLGC.

**Table 5:** Top 20 Keywords.

Rank	Keywords	Count	Centrality
1	gastric cancer	414	0.14
2	helicobacter pylori	303	0.12
3	cancer	296	0.31
4	intestinal metaplasia	292	0.13
5	precancerous lesions	195	0.15
6	risk	163	0.06
7	helicobacter pylori infection	160	0.03
8	atrophic gastritis	132	0.12
9	expression	122	0.04
10	carcinoma	53	0.11
11	stomach	51	0.00
12	diagnosis	46	0.00
13	gastric precancerous lesions	45	0.13
14	infection	43	0.07
15	classification	41	0.11
16	chronic atrophic gastritis	35	0.00
17	lesions	35	0.05
18	population	24	0.04
19	carcinogenesis	22	0.02
20	gene	19	0.05

**Figure 7:**
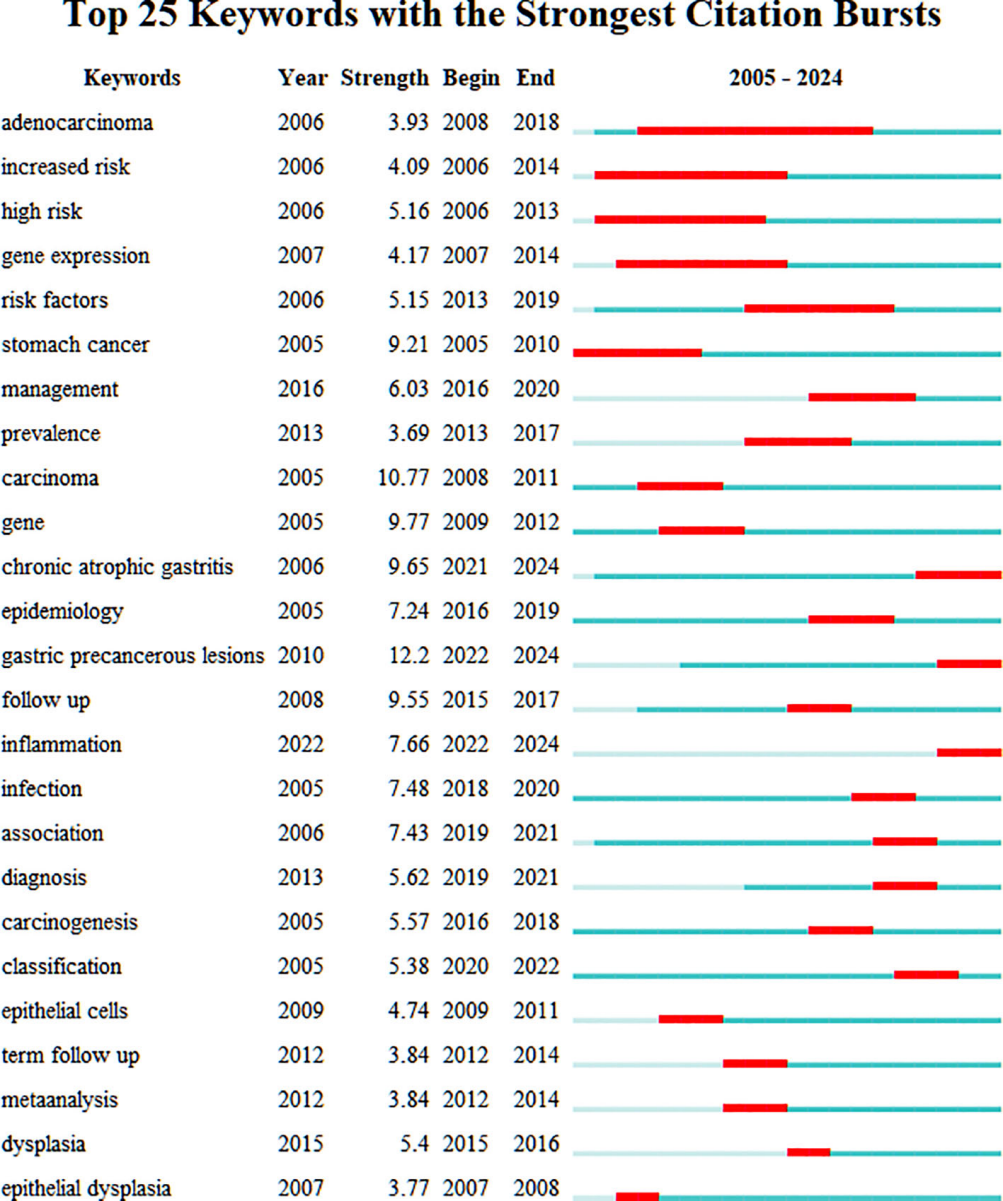
Top 25 keywords with the strongest keyword bursts.

**Figure 7** presents a burst detection analysis of keywords in the PLGC field, highlighting current research topics and trends. In this figure, red bars represent the onset, duration, and end of citation bursts. “Stomach cancer” was among the earliest keywords to emerge, with a burst strength of 9.21 lasting for six years, marking it as an initial focal topic. “Adenocarcinoma” exhibited the longest burst duration, starting in 2008 and persisting for 11 years, indicating sustained interest from researchers. Excluding keywords directly related to PLGC, “follow up” demonstrated the highest burst strength at 9.55, underscoring the importance of regular follow-up for PLGC patients. Over time, keywords such as “chronic atrophic gastritis,” “gastric precancerous lesions,” and “inflammation” have maintained prominence, reflecting their continued relevance as popular research areas in the field.

**Figure 8:**
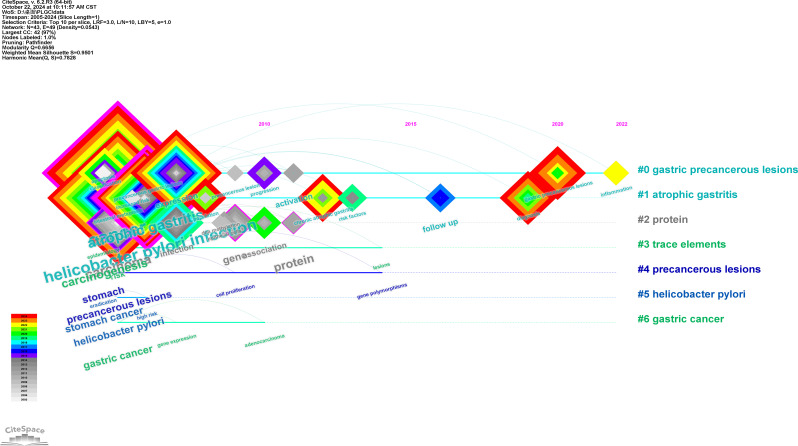
Visualization timeline view of keywords clustering analysis.

Using CiteSpace’s timeline view, keyword clusters were generated to visualize topic trends, as illustrated in **Figure 8**. The top six clusters are displayed, with each horizontal line representing a cluster of related keywords. Nodes on these lines correspond to specific keywords, and their positions indicate the year they first appeared in publications. The largest cluster was linked to “gastric precancerous lesions,” emphasizing its prominence as a primary area of focus in PLGC research.

### 3.7 Analysis of highly cited publications

**Table 6** lists the top 20 most cited articles in the field of PLGC. The most cited article is by de Vries, AC et al. (2008), titled “Gastric cancer risk in patients with premalignant gastric lesions: A nationwide cohort study in the Netherlands,” with a total of 573 citations. These highly cited articles are primarily concentrated prior to 2020, with only Song, HJ authoring two of these top articles; the rest are authored by different researchers.

**Table 6:** Top 20 cited references.

Rank	Title	Journal	Author	Year	Citation
1	Gastric cancer risk in patients with premalignant gastric lesions: A nationwide cohort study in the Netherlands	Gastroenterology	de Vries, AC	2008	573
2	Management of epithelial precancerous conditions and lesions in the stomach (MAPS II): European Society of Gastrointestinal Endoscopy (ESGE), European Helicobacter and Microbiota Study Group (EHMSG), European Society of Pathology (ESP), and Sociedade Portuguesa de Endoscopia Digestiva (SPED) guideline update 2019	Endoscopy	Pimentel-Nunes, P	2019	551
3	Management of precancerous conditions and lesions in the stomach (MAPS): guideline from the European Society of Gastrointestinal Endoscopy (ESGE), European Helicobacter Study Group (EHSG), European Society of Pathology (ESP), and the Sociedade Portuguesa de Endoscopia Digestiva (SPED)	Endoscopy	Dinis-Ribeiro, M	2012	502
4	The gastric precancerous cascade	Journal of Digestive Diseases	Correa, P	2012	480
5	Inflammation and cancer	Environmental Health and Preventive Medicine	Murata, M	2018	388
6	Carcinoma of the stomach: A review of epidemiology, pathogenesis, molecular genetics and chemoprevention	World Journal of Gastrointestinal Oncology	Nagini, S	2012	364
7	Helicobacter pylori CagA interacts with E-cadherin and deregulates the β-catenin signal that promotes intestinal transdifferentiation in gastric epithelial cells	Oncogene	Murata-Kamiya, N	2007	348
8	Randomized double-blind factorial trial of three treatments to reduce the prevalence of precancerous gastric lesions	JNCI-Journal of the National Cancer Institute	You, WC	2006	341
9	Fifteen-Year Effects of Helicobacter pylori, Garlic, and Vitamin Treatments on Gastric Cancer Incidence and Mortality	Journal of the National Cancer Institute	Ma, JL	2012	326
10	Selenium and coronary heart disease: a meta-analysis	American Journal of Clinical Nutrition	Flores-Mateo, G	2006	311
11	Gastric cancer: Prevention, screening and early diagnosis	World Journal of Gastroenterology	Pasechnikov, V	2014	306
12	Residual Embryonic Cells as Precursors of a Barrett’s-like Metaplasia	Cell	Wang, X	2011	257
13	Diagnosis of Helicobacter pylori infection: Current options and developments	World Journal of Gastroenterology	Wang, YK	2015	253
14	Overexpression of the aldo-keto reductase family protein AKR1B10 is highly correlated with smokers’ non-small cell lung carcinomas	Clinical Cancer Research	Fukumoto, S	2005	245
15	Long non-coding RNA expression profile in human gastric cancer and its clinical significances	Journal of Translational Medicine	Song, HJ	2013	201
16	Incidence of gastric cancer among patients with gastric precancerous lesions: observational cohort study in a low risk Western population	BMJ-British Medical Journal	Song, HJ	2015	186
17	Helicobacter pylori associated chronic gastritis, clinical syndromes, precancerous lesions, and pathogenesis of gastric cancer development	World Journal of Gastroenterology	Watari, J	2014	182
18	Methylation of Protocadherin 10, a Novel Tumor Suppressor, Is Associated With Poor Prognosis in Patients With Gastric Cancer	Gastroenterology	Yu, J	2009	180
19	Risk factors in gastric cancer	European Review for Medical and Pharmacological Sciences	Compare, D	2010	161
20	Helicobacter pylori eradication cannot reduce the risk of gastric cancer in patients with intestinal metaplasia and dysplasia: evidence from a meta-analysis	Gastric Cancer	Chen, HN	2016	149

**Figure 9:**
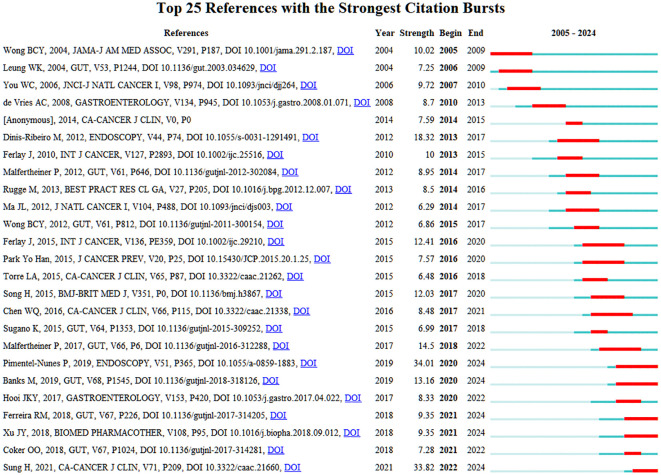
Top 25 references with the strongest citation bursts.

**Figure 9** presents a burst detection analysis of cited references in the PLGC field, highlighting specific articles and authors that have attracted substantial attention over the past two decades. In this figure, red bars represent the onset, duration, and end of citation bursts. The article with the highest burst citation is the updated management guidelines for premalignant and gastric lesions, published in Endoscopy in April 2019. These guidelines were prepared by a task force comprising members from the European Society of Gastrointestinal Endoscopy, European Helicobacter and Microbiota Study Group, European Society of Pathology, and the Society of Digestive Endoscopy (7). This article has garnered widespread attention from researchers. Another publication with a high burst strength is the 2020 Global Cancer Statistics report by GLOBOCAN, published online by the American Cancer Society in CA: A Cancer Journal for Clinicians on February 4, 2021, coinciding with World Cancer Day. This report offers updated estimates on cancer incidence and mortality across 185 countries for 36 types of cancer, highlighting the global cancer burden (8). The citation burst for this article has persisted to the present, reflecting its ongoing relevance. Other articles with sustained citation bursts include the 2019 guidelines by the British Society of Gastroenterology on the diagnosis and management of patients at risk for gastric adenocarcinoma, and a study by Rui M Ferreira et al., which explores the composition of the gastric microbiome in chronic gastritis and gastric cancer (9). Additionally, research on the Xiao Tan He Wei Decoction’s effect in reversing MNNG-induced precancerous lesions via NF-κB-mediated apoptosis has also demonstrated sustained citation bursts (10). These continued bursts demonstrate the lasting impact and current interest in these works within the PLGC research community.

### 3.8 Co-cited reference analysis

**Figure 10:**
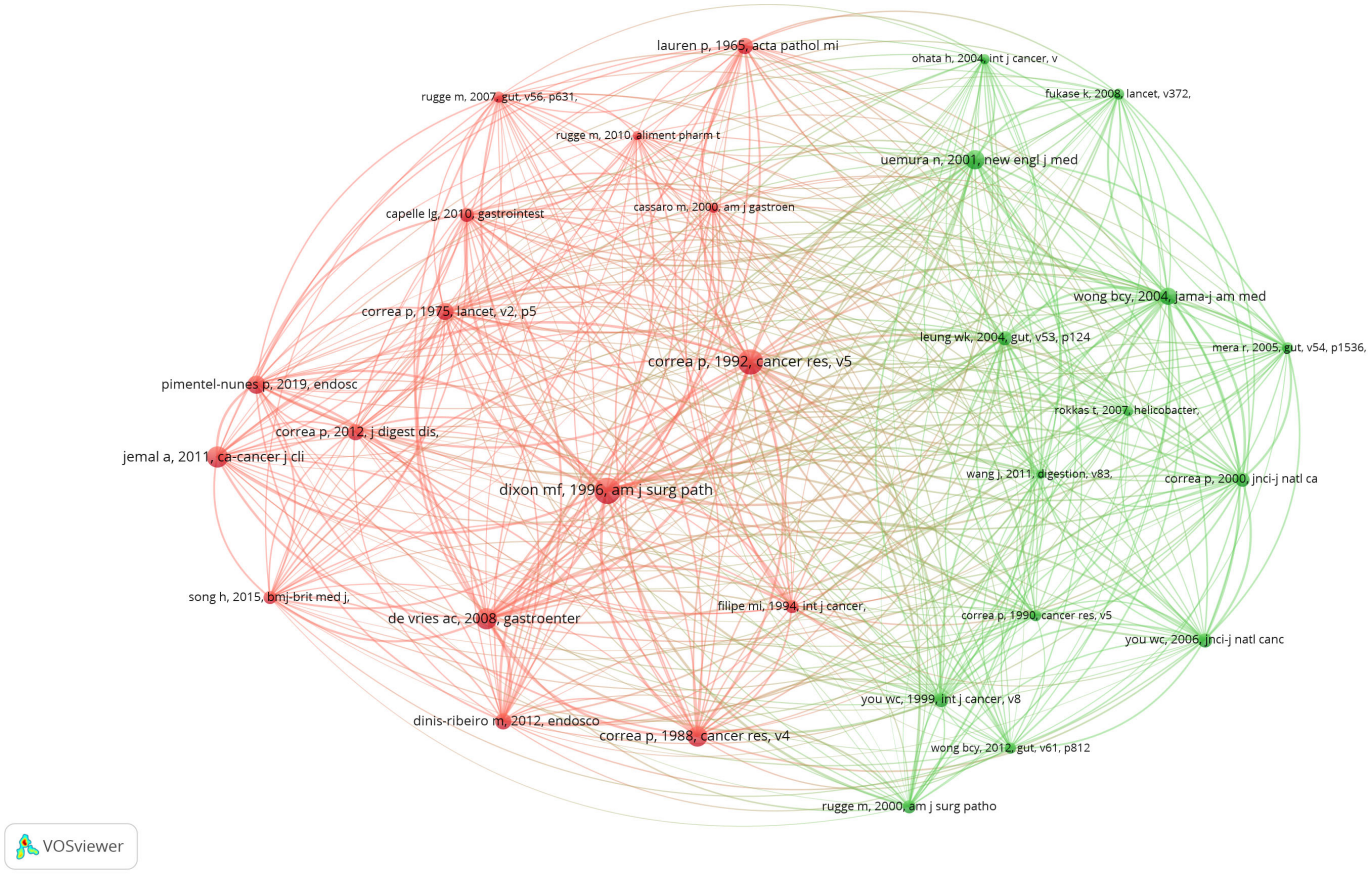
Visualization cluster view of co-cited references.

Over the past 20 years, a total of 1,141 publications have been produced on PLGC. **Table 7** lists the top 20 co-cited references, each cited at least 12 times. The most co-cited reference is the management guidelines for gastric epithelial precancerous and gastric lesions, with 103 co-citations. This high citation count highlights the broad academic recognition of the guidelines’ concepts and scoring criteria (**Table 7**). To visualize co-citation patterns, we constructed a co-citation network based on the top 30 co-cited references, as shown in **Figure 8**. In this network, the size of each node is proportional to the number of co-citations, while the lines between nodes represent co-citation relationships. The thickness and number of connecting lines indicate the strength of the linkage between citations. Key studies, such as Dixon MF (1996, Am J Surg Path), Correa P (1992, Cancer Res), and Uemura N (2001, New Engl J Med), exhibit particularly strong associations, underscoring their status as foundational and widely acknowledged works in the PLGC field (**Figure 10**).

**Table 7:** Top 20 co-cited references.

Rank	Title	Journal	Author	Year	Citation
1	Management of epithelial precancerous conditions and lesions in the stomach (MAPS II): European Society of Gastrointestinal Endoscopy (ESGE), European Helicobacter and Microbiota Study Group (EHMSG), European Society of Pathology (ESP), and Sociedade Portuguesa de Endoscopia Digestiva (SPED) guideline update 2019	Endoscopy	Pedro Pimentel-Nunes	2019	103
2	Global Cancer Statistics 2020: GLOBOCAN Estimates ofIncidence and Mortality Worldwide for 36 Cancers in 185 Countries	CA: A Cancer Journal for Clinicians	Hyuna Sung	2021	75
3	British Society of Gastroenterology guidelines on the diagnosis and management of patients at risk of gastric adenocarcinoma	Gut	Matthew Banks	2019	42
4	Management of precancerous conditions and lesions in the stomach (MAPS): guideline from the European Society of Gastrointestinal Endoscopy (ESGE), European Helicobacter Study Group (EHSG), European Society of Pathology (ESP), and the Sociedade Portuguesa de Endoscopia Digestiva (SPED)	Endoscopy	M. Dinis-Ribeiro	2012	36
5	Management of Helicobacter pylori infection—the Maastricht V/Florence Consensus Report	Gut	P Malfertheiner	2016	30
6	Gastric cancer	Lancet	Elizabeth C Smyth	2020	26
7	Cancer incidence and mortality worldwide: sources, methods and major patterns in GLOBOCAN 2012	International Journal of Cancer	J. Ferlay	2015	26
8	Helicobacter pylori Eradicationto Prevent Gastric Cancerin a High-Risk Region of China A Randomized Controlled Trial	JAMA	Benjamin Chun-Yu Wong	2004	22
9	Gastric microbial community profiling reveals a dysbiotic cancer-associated microbiota	Gut	Rui M Ferreira	2017	20
10	Incidence of gastric cancer among patients with gastric precancerous lesions: observational cohort study in a low risk Western population	BMJ	Huan Song	2015	20
11	Randomized Double-Blind Factorial Trial of Three Treatments To Reduce the Prevalence of PrecancerousGastric Lesions	Journal of the National Cancer Institute	Wei-cheng You	2006	19
12	Cancer Statistics in China, 2015	CA: A Cancer Journal for Clinicians	Wanqing Chen	2016	18
13	Estimates of worldwide burden of cancer in 2008: GLOBOCAN 2008	International Journal of Cancer	Jacques Ferlay	2010	17
14	Management of Helicobacter pylori infectiondthe Maastricht IV/Florence Consensus Report	Gut	Peter Malfertheiner	2012	17
15	Gastric Cancer Risk in Patients With Premalignant Gastric Lesions: A Nationwide Cohort Study in the Netherlands	Gastroenterology	ANNEMARIE C. DE VRIES	2008	17
16	Precancerous lesions in the stomach: from biology to clinical patient management	Best Practice & Research Clinical Gastroenterology	Massimo Rugge M.D	2013	15
17	Global Prevalence of Helicobacter pylori Infection: Systematic Review and Meta analysis	Gastroenterology	James K.Y. Hooi	2017	13
18	Mucosal microbiome dysbiosis in gastric carcinogenesis	Gut	Olabisi Oluwabukola Coker	2017	13
19	Xiao Tan He Wei Decoction reverses MNNG-induced precancerous lesions of gastric carcinoma *in vivo* and vitro: Regulation of apoptosis through NF-κB pathway	Biomedicine & Pharmacotherapy	Jingyu Xu	2018	13
20	Effects of selective COX-2 inhibitor and Helicobacter pylori eradication on precancerous gastric lesions	Gut	Benjamin C Y Wong	2012	12

## 4 Discussion

### 4.1 General information

Gastric cancer ranks as the fifth most common cancer worldwide and has long been a focal point for researchers, especially in the context of its prevention strategies (11). Gastric cancer represents the culmination of a multi-step cascade, frequently initiated by chronic inflammatory conditions such as Helicobacter pylori infection. This progressive process ultimately results in atrophic gastritis and intestinal metaplasia (12). These lesions represent the precancerous stages in the development of intestinal-type gastric cancer. PLGC are a pathological concept, referring to changes that are strongly associated with the onset of gastric cancer. The standardized diagnosis and management of PLGC are pivotal for effective gastric cancer prevention. However, most patients with gastric tumors or precancerous lesions remain asymptomatic, often leading to delayed diagnosis and treatment. As a result, there has been growing attention from researchers on these frequently overlooked and undiagnosed conditions. Topics such as risk factors, management strategies, long-term follow-up, diagnosis, and classification of PLGC have increasingly gained prominence as popular research areas across multiple disciplines (3, 13–16). This study utilizes bibliometric analysis to investigate the leading countries, institutions, researchers, and journals contributing to PLGC research. The goal is to identify popular research topics and uncover emerging trends within this field.

Since 2019, the number of publications in this field has exhibited an explosive growth trend each year. The retrieved data clearly indicates a continuously increasing research focus on PLGC, with notable contributions from China. Among the top 10 countries producing research on PLGC, eight are developed nations, while two are developing countries. China leads with the highest number of publications, followed by the USA and Japan, while Iran ranks last. As a country with a high incidence of gastric cancer, China reports approximately 679,000 new cases and 498,000 deaths related to gastric cancer annually, underscoring the urgent need for research and prevention strategies in this region (17). The incidence and mortality rates of gastric cancer increase with age, posing a severe threat to public health and contributing to substantial economic burdens. As a result, the extensive research on PLGC in China may be closely tied to these high incidence and mortality rates. However, when examining citation frequency, although China leads in the number of publications, the overall quality of these studies suggests room for improvement. This indicates that Chinese researchers still face significant challenges in achieving broader academic impact. In contrast, researchers from countries like the Netherlands and Portugal, despite producing fewer publications, demonstrate higher academic quality, reflecting greater influence and recognition in the global research community.

The institution with the highest number of publications is Peking University in China, which has contributed a total of 50 papers. Peking University also maintains strong collaborative relationships with both domestic and international institutions, reflecting its in-depth research in this field and its significant contribution to advancing PLGC research on a global scale. Among the top ten institutions by publication volume, half are based in China, underscoring the strong interest of Chinese researchers in PLGC, likely driven by the large population affected by this condition in the country. Furthermore, Chinese institutions exhibit extensive collaboration networks, enhancing the impact of their research. **Figure 4** highlights various collaborative clusters among institutions, with Peking University, Universidade do Porto, and Vanderbilt University serving as core nodes in their respective clusters. This central positioning emphasizes their leading roles in PLGC research within their networks and across the broader academic landscape.

The author with the highest number of publications is You Weicheng from the Peking University Cancer Hospital & Institute. Collectively, the top 10 authors have contributed a total of 213 papers, accounting for 18.67% of all publications on PLGC. Among them, You Weicheng, Pan Kai-Feng, and Dinis-Ribeiro Mario are recognized as key contributors in this field, each achieving relatively high average citation counts. Additionally, Correa Pelayo, Dinis-Ribeiro Mario, and Zhou Tong stand out as the authors with the highest average citation counts, reflecting the considerable academic impact of their research. These citation metrics emphasize not only the influence of individual researchers but also the growing global interest in PLGC. In summary, the authorship analysis highlights the significant contributions made by researchers from different countries in advancing PLGC research. As the most prolific author, You Weicheng has focused his work on crucial areas such as early diagnosis, treatment, and biomarker development for PLGC. His research places particular emphasis on the preventive role of Helicobacter pylori (HP) in PLGC and includes clinical trials assessing PLGC risk. These contributions underscore the importance of early intervention and risk assessment in driving progress within PLGC research (18–22).

In the analysis of publication journals, Gastroenterology stands out as the journal with the highest impact factor (IF = 25.70, Q1), featuring 12 published articles, including three high-quality clinical studies on the early detection and prognosis of PLGC. On the other hand, the journal with the highest number of publications is the World Journal of Gastroenterology (IF = 4.30, Q1), which predominantly publishes articles and reviews focusing on risk assessment of PLGC through the use of biomarkers (11, 16, 23–25). The top 20 journals by publication volume are predominantly categorized within Q1 and Q2, underscoring the considerable impact of PLGC research across relevant academic disciplines. Notably, most of these journals are affiliated with publishing institutions based in the United States, China, and the United Kingdom, which highlights the substantial contributions these countries and their journals have made to the advancement of PLGC research.

### 4.2 Hot topics and highly cited papers

In this study, we identified several significant contributions from distinguished authors whose work has made a profound impact on the PLGC research field. The use of burst detection for keywords has proven valuable in tracking research frontiers, with terms such as “chronic atrophic gastritis” and “inflammation” remaining as prominent and enduring topics. Chronic atrophic gastritis is a key histological change in PLGC, underscoring the critical need for risk management and mitigation strategies in PLGC research. Notably, the 2019 Management Guidelines for Gastric Epithelial Precancerous Conditions and Lesions, published in Endoscopy, serve as a foundational document within the field of PLGC. These guidelines emphasize that patients with chronic atrophic gastritis face an increased risk of developing gastric adenocarcinoma, thereby highlighting the indispensable role of endoscopic monitoring in the management and prevention of chronic atrophic gastritis. Screening and surveillance of at-risk populations are critical strategies for reducing gastric cancer mortality through early detection and timely treatment. For patients with advanced chronic atrophic gastritis, high-quality endoscopic follow-up is recommended every three years. Additionally, individuals with focal intestinal metaplasia, incomplete intestinal metaplasia, persistent Helicobacter pylori gastritis, or a family history of gastric cancer are advised to undergo chromoendoscopy, accompanied by biopsy monitoring, within a three-year period. These measures play a vital role in identifying precancerous changes and managing gastric cancer risk effectively (7).Additionally, identifying and monitoring PLGC in high-risk regions for gastric cancer is considered a cost-effective strategy for reducing disease burden. However, this approach also presents challenges, including potential adverse reactions and the growing concern of antibiotic resistance, particularly in the management of Helicobacter pylori infections. Addressing these issues is essential for optimizing prevention and surveillance programs in such regions (11).

Single-cell transcriptome analysis has unveiled new insights into the biological characteristics of gastric cancer, offering a deeper understanding of its molecular heterogeneity and cellular dynamics. This advanced technique enables the identification of distinct cellular populations and key gene expression profiles within the tumor microenvironment, revealing crucial mechanisms involved in cancer progression, immune evasion, and therapeutic resistance. Such findings hold significant promise for improving personalized treatment strategies and advancing the development of targeted therapies in gastric cancer research (26). As a distinct model of intratumoral heterogeneity, gastric cancer continues to lack a comprehensive understanding of the specific cell types and microenvironments that drive its carcinogenic processes. Despite advances in molecular and cellular research, the complex interactions within the tumor microenvironment, including immune cells, stromal components, and tumor-cell subsets, remain insufficiently explored. An in-depth investigation into these aspects is essential for elucidating the mechanisms of gastric cancer progression, paving the way for the development of more effective diagnostic tools and targeted therapeutic strategies (27). Through RNA sequencing, Kim, J, and colleagues conducted a comprehensive single-cell analysis of heterogeneous cell populations within both precancerous lesions and gastric cancer, uncovering critical insights into cellular dynamics and differentiation pathways. They identified ten distinct subtypes of gastric cancer cells and highlighted key cellular differences between intestinal-type and diffuse-type gastric cancer. Their findings indicate that intestinal-type cancer cells differentiate along the intestinal metaplasia lineage, while diffuse-type cancer cells exhibit traits reminiscent of *de novo* differentiation. Furthermore, they discovered an enrichment of CCND1 mutations within precancerous lesions and demonstrated that cancer-associated fibroblasts possess stem-cell-promoting properties, underscoring their role in tumor progression. Notably, tumor cells were categorized into previously established molecular subtypes, with specific malignant cell subtypes showing high levels of epithelial-to-mesenchymal transition (EMT). This EMT was strongly associated with poor clinical outcomes, offering a potential marker for prognosis. Beyond intratumoral heterogeneity, their analysis also illuminated the roles of various cellular lineages in driving potential carcinogenic pathways, providing a deeper understanding of the interplay between the tumor microenvironment and cancer development. These findings contribute significantly to the field of gastric cancer research and may guide future therapeutic strategies (28). Single-cell transcriptome studies of gastric precancerous lesions and gastric cancer offer critical insights into the behavior of gastric cancer cells, suggesting potential diagnostic and therapeutic targets for improved patient outcomes (28).

HP infection is a well-established pathogenic factor for PLGC. An updated report from a 26.5-year follow-up randomized controlled trial, conducted by Fujian Medical University, evaluated the long-term effects of HP eradication therapy on gastric cancer incidence and mortality in high-risk populations. Initiated in July 1994 in high-risk regions of southern China, the study revealed that participants receiving HP eradication treatment had a significantly lower risk of developing gastric cancer compared to those in the placebo group. This risk reduction was particularly pronounced among individuals without precancerous lesions or dyspeptic symptoms at baseline. Specifically, in the placebo group of 527 participants with persistent HP infection, 32 cases of gastric cancer were observed. By contrast, among the 625 participants in the treatment group who successfully eradicated HP, only 16 cases of gastric cancer were identified. These findings strongly suggest that HP eradication therapy provides long-term protective effects against gastric cancer, especially for infected individuals without baseline precancerous lesions in high-risk populations (29).

Pasechnikov, V, and colleagues reviewed advances in the prevention, screening, and early diagnosis of gastric cancer, emphasizing the challenge that many cases are still diagnosed at advanced stages, leading to poor outcomes. They noted that interventions targeting HP—a major pathogenic factor—remain insufficient, despite the cost-effectiveness of large-scale eradication programs in high-risk regions. Concerns about side effects and antibiotic resistance persist. Prevention strategies are categorized into primary and secondary approaches. Primary prevention includes avoiding carcinogens, strengthening host defenses, lifestyle adjustments, and using antioxidants. Secondary prevention focuses on screening and managing PLGC, with a strong emphasis on follow-up during this stage. Molecular biology-based tools, such as microRNAs, cancer autoantibodies, and volatile biomarkers, have gained attention as diagnostic aids, though limited association strength hinders their adoption. Endoscopic training and experience are pivotal for effective screening, with standardized imaging and thorough preparation essential, as endoscopy remains the gold standard for early diagnosis (11).

Inflammation plays a critical role in carcinogenesis, with chronic inflammation linked to an elevated risk of various malignancies. For example, HP infection induces chronic inflammation, which can drive the development of gastric cancer. Invasive gastric cancer is often preceded by a series of precancerous lesions. The progression begins with acute inflammation, followed by chronic active inflammation, which may persist as non-atrophic gastritis without glandular loss, eventually advancing to multifocal atrophic gastritis. Research suggests that patients with HP infection exhibit significantly higher levels of 8-nitroguanine and 8-oxodG in gastric glandular epithelium compared to uninfected individuals. Additionally, there is a marked accumulation of PCNA, a prognostic marker for gastric cancer, in gastric epithelial cells. This sequential process, connected to gastric cancer development, is referred to as the “gastric precancerous cascade,” reflecting a dynamic interplay of inflammation, oxidative damage, and cellular proliferation (30).

Temporal clustering analysis identified several PLGC-related hotspots, with “protein” emerging as a key topic. Proteomics, the comprehensive study of protein composition and regulation, examines protein expression, modifications, and interactions to uncover the dynamics of protein networks. This approach provides critical insights into the molecular mechanisms driving PLGC progression and offers potential avenues for biomarker discovery and targeted therapy development (31, 32). In 2014, a study performed proteomic analysis on serum samples from individuals with precancerous gastric lesions, gastric cancer, and healthy controls. The primary goal was to construct a diagnostic model for detecting PLGC and gastric cancer. However, the study’s small sample size raised concerns about its reliability, highlighting the need for further validation with larger cohorts to ensure robustness and clinical applicability (33). In 2021, Peking University conducted a tissue-based proteomic analysis involving 324 subjects. The study began with a case-control analysis in high-risk gastric cancer regions in China, followed by cohort studies, prospective follow-ups, and validation through independent case-control studies. Significant proteomic differences between PLGC and gastric cancer were identified. Importantly, the integration of four specific proteomic markers—APOA1BP, PGC, HPX, and DDT—greatly improved the prediction of disease progression. These findings were validated through immunohistochemistry and mRNA testing, demonstrating translational significance for identifying high-risk PLGC populations and enhancing early detection of gastric cancer. This research may contribute to targeted prevention and timely intervention strategies (34).

Traditional Chinese Medicine (TCM) suggests that tongue characteristics—including color, size, shape, coating color, thickness, and moisture—reflect overall health. Building on this principle, Yuan, L., and colleagues developed a machine learning tool leveraging tongue images to diagnose PLGC. The study utilized tongue images and coating microbiome samples, creating a deep learning model to assess their diagnostic value in detecting precancerous gastric lesions. Conducted in two phases, the study first analyzed samples from 328 newly diagnosed gastric cancer patients and 304 non-gastric cancer participants. Subsequently, multi-center validation expanded the sample size to 937 gastric cancer patients and 1,911 non-gastric cancer participants, with independent external validation. The tool achieved an AUC value of 0.89, consistently demonstrating strong predictive performance across all stages. This strategy significantly outperformed traditional blood biomarker combinations, showcasing its potential for PLGC diagnosis and early detection (35).

In recent years, the relationship between PLGC and the gut microbiome has become a key research focus, revealing the critical role of the microbiome in cancer onset and progression. Changes in the gastrointestinal microbiome not only affect cancer susceptibility but may also promote disease progression by shaping the tumor microenvironment and regulating host immune responses. Studies have shown that gastric microbiome dysbiosis, such as Helicobacter pylori infection, is closely associated with gastric cancer development, while certain probiotics, such as Lactobacillus and Bifidobacterium, exhibit anti-inflammatory and anti-cancer potential by enhancing immune surveillance and improving the tumor microenvironment (36). Additionally, shifts in the microbiome can serve as early warning signals for PLGC, providing valuable insights for early diagnosis. The role of the gut microbiome in the development of PLGC and gastric cancer is also gaining attention, with dysbiosis believed to facilitate immune evasion and cancer development. Animal model studies have demonstrated a significant correlation between changes in the gut microbiome and the progression of PLGC, offering new perspectives for microbiome-based interventions (37). Meanwhile, TCM has shown potential efficacy in regulating the microbiome, improving microbial balance, and promoting the growth of beneficial bacteria, thus opening new avenues for PLGC treatment. These findings highlight the clinical value of microbiome-based interventions and immunotherapy, such as the application of probiotics or combined microbiome regulation and immunotherapy. However, further exploration of how specific microbial communities influence PLGC and its complex interactions with host immune responses remains a priority for future research (38).

Immunotherapy has emerged as a key focus in the treatment of PLGC and gastric cancer, offering new opportunities for clinical practice. The immune checkpoint molecule PD-L1 is highly expressed in gastric cancer patients, and polymorphisms in its promoter region are closely associated with gene transcription activity and tumor immune evasion. Studies have revealed that the transcription factor SP1 is a critical binding protein in the PD-L1 promoter region, facilitating PD-L1 overexpression and enhancing tumor cell immune evasion. This mechanism has not only deepened the understanding of PD-L1’s role in the progression of PLGC and gastric cancer but also laid the foundation for developing PD-L1-based immunotherapeutic strategies (39). At the same time, the clinical application of immunotherapy is advancing towards personalized treatment, emphasizing the precision of therapeutic approaches and the clear identification of indications. Combining immunotherapy with other treatment modalities, such as chemotherapy, targeted therapy, or microbiome modulation, has become a pivotal strategy for improving efficacy. Future research must further optimize the safety of immunotherapy, identify biomarkers for precision treatment, and explore the synergistic effects of different therapeutic approaches. These advances are expected to drive innovation in PLGC and gastric cancer treatment plans, providing patients with more effective clinical options (40).

## 5 Conclusion

This bibliometric analysis provides a comprehensive overview of the research landscape and evolving trends in PLGC from 2005 to 2024. The study reveals a steady annual increase in publications, with China leading in output, while countries such as the Netherlands and Portugal demonstrate higher academic impact per publication. Key institutions, including Peking University and Vanderbilt University, as well as prolific authors like You Weicheng and Dinis-Ribeiro Mario, underscore the collaborative and interdisciplinary nature of PLGC research. High-frequency keywords, such as Helicobacter pylori, intestinal metaplasia, and chronic atrophic gastritis, highlight the central role of microbial infection, histological progression, and inflammation in PLGC pathogenesis. Burst detection and co-citation analyses identify emerging research frontiers, including single-cell transcriptomics, proteomics, and machine learning applications in early diagnosis, while sustained interest remains in guideline-driven management and risk stratification. Mechanistic studies on molecular pathways (e.g., NF-κB-mediated apoptosis) and clinical innovations in endoscopic surveillance continue to play a crucial role in advancing PLGC prevention.

However, this study has certain limitations that warrant further exploration. For instance, the reliance on data from a single database may introduce potential biases, such as incomplete coverage of relevant publications or regional disparities. Additionally, the bibliometric methods employed, while effective for mapping research trends, may not capture the nuanced details of experimental findings or clinical outcomes. Future studies should aim to address these limitations by validating findings across more comprehensive databases, such as PubMed and Scopus, to ensure broader coverage and minimize bias. Moreover, adopting more advanced analytical techniques, such as artificial intelligence-driven bibliometric tools, could facilitate deeper insights into PLGC mechanisms and clinical applications. Expanding international collaboration and integrating multidisciplinary approaches will also be critical for bridging gaps in early detection, risk stratification, and therapeutic intervention. These efforts are essential for reducing the global burden of gastric cancer and advancing the field of PLGC research.

## Data Availability

The raw data supporting the conclusions of this article will be made available by the authors, without undue reservation.
